# How Does cGAS Avoid Sensing Self-DNA under Normal Physiological Conditions?

**DOI:** 10.3390/ijms241914738

**Published:** 2023-09-29

**Authors:** Wangli Zheng, Nanhua Chen, François Meurens, Wanglong Zheng, Jianzhong Zhu

**Affiliations:** 1 College Veterinary Medicine, Yangzhou University, Yangzhou 225009, China; wanglizhengg@163.com (W.Z.); hnchen@yzu.edu.cn (N.C.); 2 Joint International Research Laboratory of Agriculture and Agri-Product Safety, Yangzhou University, Yangzhou 225009, China; 3 Comparative Medicine Research Institute, Yangzhou University, Yangzhou 225009, China; 4 Jiangsu Co-Innovation Center for Prevention and Control of Important Animal Infectious Diseases and Zoonoses, Yangzhou 225009, China; 5 Swine and Poultry Infectious Diseases Research Center, Faculty of Veterinary Medicine, University of Montreal, St. Hyacinthe, QC J2S 2M2, Canada; francois.meurens@umontreal.ca; 6 Department of Veterinary Microbiology and Immunology, Western College of Veterinary Medicine, University of Saskatchewan, Saskatoon, SK S7N 5E2, Canada

**Keywords:** cGAS, self-DNA, mitosis, autoimmune diseases, DNases

## Abstract

cGAS is a cytosolic DNA sensor that activates innate immune responses by producing the second messenger 2′3′-cGAMP, which activates the adaptor STING. cGAS senses dsDNA in a length-dependent but sequence-independent manner, meaning it cannot discriminate self-DNA from foreign DNA. In normal physiological conditions, cellular DNA is sequestered in the nucleus by a nuclear envelope and in mitochondria by a mitochondrial membrane. When self-DNA leaks into the cytosol during cellular stress or mitosis, the cGAS can be exposed to self-DNA and activated. Recently, many studies have investigated how cGAS keeps inactive and avoids being aberrantly activated by self-DNA. Thus, this narrative review aims to summarize the mechanisms by which cGAS avoids sensing self-DNA under normal physiological conditions.

## 1. Introduction

Innate immunity is the first immunological, non-specific mechanism for combating infections and other aggressions. The initial sensing of infection and injury is mediated by pattern recognition receptors (PRRs), which recognize pathogen-associated molecular patterns (PAMPs) and damage-associated molecular patterns (DAMPs). Excessively accumulated cytosolic DNA can act as a PAMP or DAMP, and the presence of DNA in the cytoplasm is normally a sign of microbial infection or tissue damage [1]. The presence of naked DNA in the cytoplasm of mammalian cells triggers a cellular response initiated by the DNA sensing pathway. Recently, cyclic GMP-AMP synthase (cGAS) was characterized as a primary cytosolic DNA sensor that triggers type I interferons (IFNs) and inflammatory cytokines upon binding dsDNA [2,3].

cGAS can be activated by dsDNA in a length-dependent but sequence-independent manner by binding the DNA phosphate backbone [4]. Thus, cGAS is activated not only by non-self DNA, such as DNA from DNA viruses or retroviruses, intracellular bacteria, and protozoa but also by self-DNA, including mitochondrial and nuclear DNA that gain access to the cytoplasm. When self-DNA leaks into the cytoplasm during cellular stress (such as mitochondrial alteration, DNA damage, mitotic arrest, or senescence) or is present as cytosolic micronuclei, cGAS is activated leading to a state of sterile inflammation [5,6]. This protective component of innate immunity against microbial infection will result in autoimmune diseases if it unexpectedly comes into contact with self-DNA [7].

In normal physiological conditions, cellular DNA is isolated in the nucleus and mitochondria through the nuclear envelope and the mitochondrial membrane, respectively [8]. However, the nuclear envelope is a highly dynamic structure that is disassembled and reassembled during mitosis in higher eukaryotes [9]. Unsurprisingly, the cGAS could be directly exposed to self-DNA during mitosis when the nuclear envelope is disassembled. This has raised a question: When cGAS is exposed to self-DNA, how does it keep inactive? Thus, the aim of this narrative review is to summarize the mechanisms by which cGAS avoids sensing self-DNA and keeps inactive under normal physiological conditions.

## 2. Structural Domains and Modification Sites of cGAS

### 2.1. Structural Domains of cGAS

cGAS (also known as C6orf150 encoded by MB21D1) is located on chromosome 6q13 and composed of 522 amino acids in human [10]. The cGAS protein (human) is composed of an unstructured and not well-conserved N-terminus (1–160) and a highly conserved C terminus (161–522) that contains a nucleotidyltransferase (NTase) core domain (161–330) and the male abnormal 21 (Mab21) domain (213–513) [11]. By comparing the amino acid sequences of cGAS in different mammalian species, it is found that the N-terminal domain (1–160) of cGAS is diverse and disordered [12]. This less evolutionarily conserved sequence exerts a critical role in sensing nuclear chromatin, binding to immune-stimulatory DNA (ISD), cytoplasmic distribution, determining nuclear plasma membrane, and assembly of lipid phase condensation [13]. Recent studies have shown that the serine (13, 37, 64, 129 and 143) residue in the cGAS N-terminus is crucial for sensing genomic/chromatin DNA [14]. The C-terminal NTase domain contains several conserved amino acid residues within the NTase superfamily, including G212, S213, E225, D227 and D319, which are critical for the enzyme activity of cGAS [14]. The Mab21 domain harbors the conserved Zn finger motif (H390-C405), which is functionally important to scale the specificity of cGAS toward dsDNA [12].

### 2.2. Transcriptional and Epigenetic Regulation of cGAS

IFN could induce the gene encoding of cGAS, which is a positive feedback for amplification of the cGAS pathway [15]. The promoter of cGAS contains one STAT1 binding site and three IFN-sensitive response elements (ISREs), by which the synthesis of cGAS could regulated by IFN [12]. It has indicated that at the early stage of viral DNA activation, the induction of cGAS is IFNAR-dependent but IRF7-independent, and deleting the gene of IFNAR1 or STAT1 could suppress the induction of cGAS [16]. The study has suggested that the first wave of IFN-I exerts a critical role in subsequent positive feedback regulation of DNA-triggered IFN-I production [16]. Furthermore, it was identified that the transcription factors Sp1 or CREB participated in regulating the human cGAS gene [17]. The promoter activity of cGAS could be significantly improved by the over-expression of Sp1 or CREB and markedly restrained by the knocking-down of endogenous Sp1 and CREB [17]. Additionally, due to the loss of the expression of cGAS, several tumor cell lines are defective in producing IFNs and cytokines after stimulating by DNA or infecting by viruses [15]. In some cases, the suppression of cGAS expression could be partially reversed by using inhibitors of DNA methylation, which indicates that the gene encoding of cGAS was regulated by epigenetic mechanisms [15].

### 2.3. Post-Translational Modifications of cGAS

**Figure 1:**
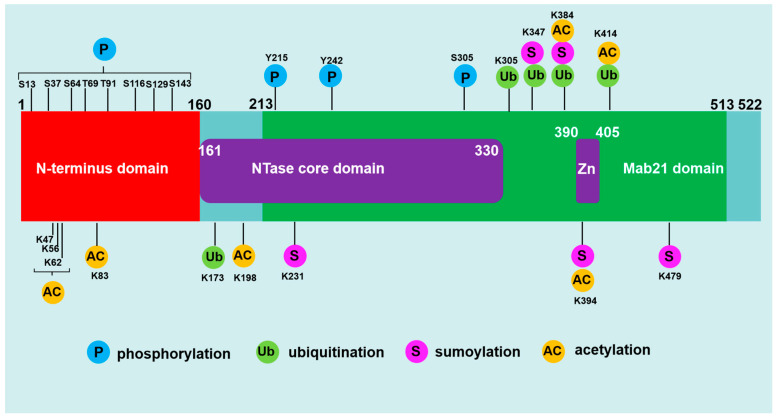
Sequence structure and post-translational modifications of cGAS. (1) Human cGAS is a protein consisting of 522 amino acids. It is composed of an N-terminus (1–160) and a C terminus (161–522) that contains a nucleotidyltransferase (NTase) core domain (161–330) and the male abnormal 21 (Mab21) domain (213–513). The Mab21 domain harbors the conserved Zn finger motif (390–405). (2) cGAS is regulated by various post-translational modifications, and the phosphorylation, ubiquitination, sumoylation and acetylation are major post-translational modifications in the cellular cGAS. (3) The common post-translational modification sites of cGAS are illustrated here. Human cGAS could be hyperphosphorylated at serine, threonine, and tyrosine residues, including Ser13, Ser37, Ser64, Ser116, Ser129, Ser143, Thr69, Thr91, Tyr215 and Tyr242. Human cGAS could be ubiquitinated at Lys-173, Lys-335, Lys-347, Lys-384 and Lys414. Human cGAS could be sumoylated at Lys231, Lys479, Lys-347, Lys-384 and Lys-394. Human cGAS could be acetylated at Lys47, Lys56, Lys62, Lys83, Lys198, Lys384, Lys394, and Lys414.

The protein of cGAS contains many modification sites, which can be regulated by various post-translational modifications, including phosphorylation, ubiquitination, sumoylation, and acetylation [18] (Figure 1). Recently, several studies have demonstrated that cGAS could be hyperphosphorylated in humans at serine, threonine and tyrosine residues such as Ser13, Ser37, Ser64, Ser116, Ser129, Ser143, Thr69, Thr91, Tyr215 and Tyr242 by phosphorylase kinases among which serine/threonine-protein kinase (AKT), cyclin-dependent kinase-1 (CDK1), and Aurora kinase B (AKB) and B lymphocyte kinase (BLK) are typical ones [12,19]. Ubiquitination and deubiquitination have additional control over cGAS activation [20]. Several ubiquitin ligases including RNF185, RINCK, TRIM56, ARIH1 and MARCH8 were shown to catalyze human cGAS at Lys-173, Lys335, Lys-347, Lys-384 and Lys414 respectively [21,22]. Sumoylation also plays a critical role in regulating the activity of cGAS [23]. TRIM38 maintains the sumoylation of Lys231 and Lys479 in human cGAS, which prevents K48-linked cGAS polyubiquitination and degradation [23]. Sumoylation at Lys-347, Lys-384 and Lys-394 prevents DNA-binding, oligomerization and nucleotidyltransferase activity [18]. Depending on the acetylation site, the acetylation of cGAS can positively or negatively regulate the activity of cGAS [24,25]. Human cGAS was reported to be acetylated at Lys47, Lys56, Lys62, Lys83, Lys198, Lys384, Lys394, and Lys414 [24].

## 3. How Is the cGAS Activated

### 3.1. DNA-Induced Conformational Changes in cGAS Lead to Its Activation

cGAS activation requires direct binding to dsDNA to form cGAS-dsDNA complexes, within which the catalytic site of cGAS is structurally rearranged to activate its enzymatic activity to synthesize 2′3′-cGAMP [26]. Employing a positively charged surface and the zinc thumb dimerization domain to interact with the DNA sugar-phosphate backbone, cGAS forms extensive electrostatic interactions and hydrogen bonds with dsDNA. GAS can be activated by dsDNA in a length-dependent but sequence-independent manner [27]. The structure of the cGAS-DNA complex shows that the interaction interfaces cover about 10 bp on dsDNA [28]. Biochemical assays have demonstrated that short DNAs of <~20 bp can bind to cGAS, while longer dsDNAs of >45 bp can form more stable ladder-like networks of cGAS dimers, leading to stronger enzymatic activity [28].

cGAS alone is monomeric and does not have catalytic activity due to local structure destabilization of the NTase domain. DNA binding induces conformational changes in cGAS and crosslinks two cGAS molecules to form a 2:2 dimer or higher-order complexes, resulting in the activation of cGAS. cGAS has three DNA binding sites (A, B, and C) that interact with the sugar-phosphate backbone of dsDNA or RNA–DNA hydrides in a sequence-independent pattern [2]. Site A is the primary site mediates DNA-induced conformational change of the activation loop in cGAS. Sites A and B together mediate the formation of the 2:2 cGAS-DNA complex. Site C provides an additional interaction between cGAS and DNA, contributing to the phase separation of the cGAS-DNA complex [29]. Interestingly, although ssDNA and dsRNA can bind cGAS, both fail to rearrange the cGAS catalytic pocket, which is indispensable for cGAS activation [30].

### 3.2. Liquid-Liquid Phase Separation Can Enhance the Activation of cGAS

Liquid-liquid phase separation (LLPS) is a process by which bio-macromolecules, particularly proteins, condense into a dense phase that resembles liquid droplets [31]. LLPS is a concentration and environment-dependent condensation process driven by solute–solute interactions that energetically overcome solute–solvent interactions [32]. In a liquid-like state, the condensed phase frequently exchanges materials with the dilute phase, and this liquid-like property has an important role in defining the composition and biochemical activity of molecules in the condensed phase [32].

Both in vitro and in vivo studies have shown that interactions between cGAS and DNA result in high-order oligomerization and formation of LLPS [33]. DNA binding to cGAS induces a robust phase transition to liquid-like droplets, which could serve as a micro-reactor to accelerate 2′3′-cGAMP production by increasing local concentrations of proteins and reactants [33,34]. Additionally, cGAS forms liquid-like condensates with double-stranded DNA (dsDNA) to enhance the production of 2′3′-cyclic GMP–AMP (cGAMP) by protecting DNA from degradation by the exonuclease TREX1 [35]. This mechanism allows cGAS to detect the presence of DNA in the cytoplasm above a certain threshold to trigger a switch-like response. Further studies also demonstrated that mutation and truncation of cGAS and short DNA (<45 bp) attenuate the oligomerization and LLPS of cGAS-DNA, resulting in reduced or even eliminated cGAS activity [33,36].

### 3.3. Divalent Cations Substantially Promote the Activity of cGAS

Metal ions are essential for the functionality of a plethora of proteins [37]. About one-third of all known enzymes require particular metal co-factors for their catalytic, structural, or regulatory functions [37]. cGAS belongs to the NTase superfamily, which catalyzes nucleophilic substitution reactions and is divalent cation-dependent. The catalytic core structures of different NTases usually share a common structural fold and similar active sites harboring a highly conserved catalytic triad hG[GS], [DE]h[DE]h, h[DE]h (where h indicates a hydrophobic amino acid) for catalytic metal coordination [38]. Extensive ionic interactions formed by the positively charged surfaces of cGAS and negatively charged DNA are responsible for the combination of cGAS and DNA [33]. Such interactions are vulnerable to cytoplasmic salt concentrations. cGAS enzyme activity was much weaker in an assay with a physiological buffer than in an assay with a low-salt buffer [33]. Several studies have shown that divalent metal ions, including zinc (Zn^2+^), manganese (Mn^2+^), and magnesium (Mg^2+^), all play essential roles in regulating the activation of cGAS [39,40].

In the presence of cytosolic DNA, Zn^2+^ could facilitate cGAS activation in cells by promoting cGAS phase transition [39]. Through measurements of free Zn^2+^ concentrations, it is revealed that the molecule that Zn^2+^ binds to is cGAS but not DNA. When L929 cells were depleted of zinc with the zinc-specific chelator TPEN, the production of 2′3′-cGAMP was decreased after transfecting with HT-DNA, and this phenomenon was gradually intensified as an increasing amount of TPEN was added [33]. Furthermore, a study has indicated that Mn^2+^ could directly activate the cGAS, in which case DNA is dispensable [38]. Structural analysis revealed that Mn^2+^-activated cGAS undergoes globally similar conformational changes to DNA-activated cGAS, and forms a unique helix to widen the catalytic pocket, allowing substrate entry and 2′3′-cGAMP synthesis [38]. Further, Mn^2+^ improves the sensitivity of cGAS to intracellular DNA and its catalytic activity to promote cGAS to generate 2′3′-cGAMP under the stimulation of low concentration of DNA [40]. Another evidence is that Mn-deficient mice produced decreased amounts of cytokines and were more vulnerable to DNA viruses [38]. Structural studies of ternary complexes of dsDNA-bound cGAS have shown that the triphosphate moieties of ATP and 5′-pppG(2′,5′)pG were coordinated to Mg^2+^ [38]. Incubation of human cGAS protein with ATP, GTP, Mg^2+^, and dsDNA could result in the induction of 2′3′-cGAMP [38].

## 4. How Does cGAS Avoid Sensing Self-DNA under Normal Conditions?

### 4.1. Self-DNA Is Cleared by the DNases

The function of deoxyribonucleases (DNases) has been suggested to degrade self-DNA under normal conditions, preventing aberrant activation of cGAS-mediated immune responses [41]. DNases maintain the level of cytosolic DNA under the threshold of cGAS activation to retain immune silence. Three DNases, including DNase I, DNase II, and TREX1 (or DNase III), have been identified to degrade self-double-stranded DNA (dsDNA). DNase I is localized in serum, where it degrades the chromatin released from dead cells and prevents autoimmune diseases [42]. DNase II is localized in the lysosomes and is largely responsible for the clearance of DNA from dead cells and expelled nuclei [43]. TREX1, a major mammalian 3′-5′ DNA-exonuclease, floats in the cytosol and prevents endogenous DNA accumulation [44].

**Figure 2:**
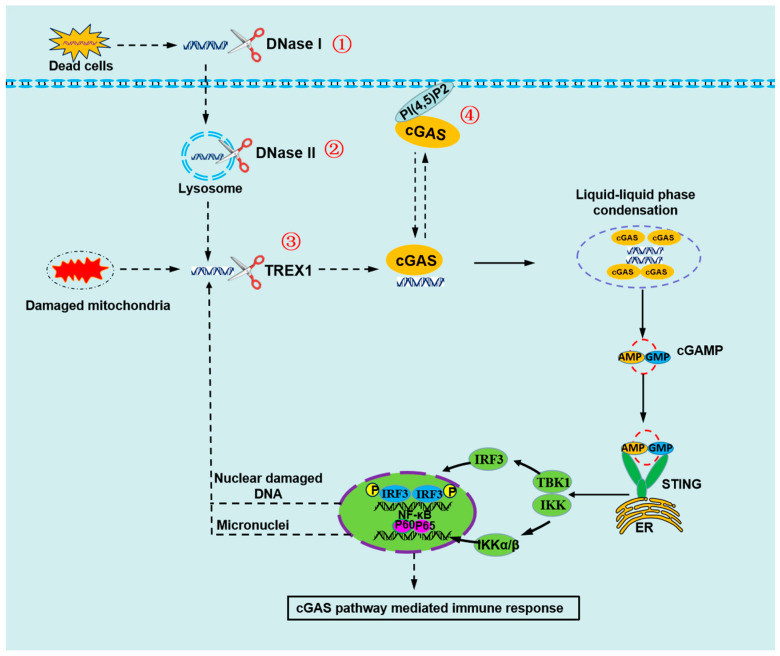
The mechanisms by cGAS to avoid sensing self-DNA during interphase. ① Self-DNA in serum or on cell surface from the dead cell is degraded by DNase I. Cell-free chromatin particles released from the billions of cells that die in the body daily can enter into healthy cells and induce innate immune responses. DNase I is localized in serum, where it degrades the chromatin released from dead cells and prevents autoimmune diseases. ② Self-DNA in endosome or lysosome is degraded by DNase II. Endosomes and lysosomes are membrane-bound organelles crucial for the normal functioning of the eukaryotic cell. Self-DNA from dead cells and expelled nuclei can be delivered into lysosomes by endocytosis and phagocytosis. DNase II is localized in the lysosomes and is largely responsible for the clearance of DNA. ③ Self-DNA in cytoplasm is degraded by TREX1. TREX1, localized in the cytosol, is a powerful DNA-degrading enzyme. Cytosolic DNA could be degraded by TREX1, which could prevent inappropriate innate immune activation. ④ cGAS is located in the plasma membrane through interacting with PI(4,5)P2, which is least likely to detect self-DNA and prevent aberrant activation. The N-terminus of cGAS in humans has a phosphoinositide-binding domain and interacts selectively with phosphatidylinositol 4,5-bisphosphate (PI(4,5)P2) in the plasma membrane.

Dysfunction of DNases can lead to the accumulation of DNA in the cytoplasm and promote the activation of cGAS-mediated immune responses (Figure 2). Loss-of-function mutations of TREX1 have been identified in human patients with autoimmune disorders such as Aicardi-Goutières syndrome (AGS) and lupus-like autoimmune disorders [45,46]. It has been suggested that cGAS pathway activation is the key signaling for autoimmunity diseases caused by a TREX1 missense mutation [45]. A study has indicated that the deficiency of TREX1 could result in an aberrant accumulation of self-DNA in the cytosol, which could predominantly activate the innate immune response via the cGAS pathways [47]. Another in vivo study has proved that genetic deletion of Trex1 or DNase II could lead to lethal autoimmune diseases in mice [48].

### 4.2. Plasma Membrane Localization of cGAS Prevents Recognition of Self-DNA

cGAS was initially thought to diffuse throughout the cytosol, waiting for or searching its DNA ligand. Recently, scientists have developed a new model that cGAS is not a cytosolic protein but a peripheral membrane protein primarily residing on the plasma membrane [49]. Analysis of the distribution of cGAS in human THP1 monocyte provided proof for the viewpoint that cGAS resides primarily at the cytoplasmic membrane of THP1 cells in its inactive state [49]. Within 30 min of DNA transfection, cGAS was no longer concentrated at the cell surface but was rather detected in various cytoplasmic puncta [49]. The unstructured N-terminus may play a role in cGAS plasma membrane attachment that contributes to restraining cGAS activation [49]. It has shown that cGAS attached to the plasma membrane through the phosphoinositide-binding domain of the N-terminus interacting with phosphatidylinositol 4,5-bisphosphate (PI(4,5)P2) [49]. The amino acids 64 to 75 of cGAS are important for its plasma membrane localization, and within this region are two arginine residues that are conserved or charge-conserved among humans, mice, and several other mammalian species [49].

Plasma membrane localization is an important way that cGAS avoids recognizing self-DNA. In resting cells, its N terminus positions cGAS at the cell surface, where it is least likely to detect self-DNA and thus prevent aberrant activation [49]. Upon DNA detection, the N-terminus may release from PI(4,5)P2, facilitating liquid droplet formation and signaling in the cytosol [49]. The N-terminal domain of cGAS is necessary and sufficient for cGAS localization to the plasma membrane, and this interaction restrains cGAS activity from self-DNA [49,50]. Deleting the N-terminal localization domain from cGAS leads to heightened sensitivity to genotoxic stress [51]. Thus, localizing to the plasma membrane is an excellent strategy for cGAS to avoid inappropriate self-DNA detection (Figure 2).

### 4.3. Binding to Histones Prevents cGAS from Sensing Self-DNA during Mitosis

**Figure 3:**
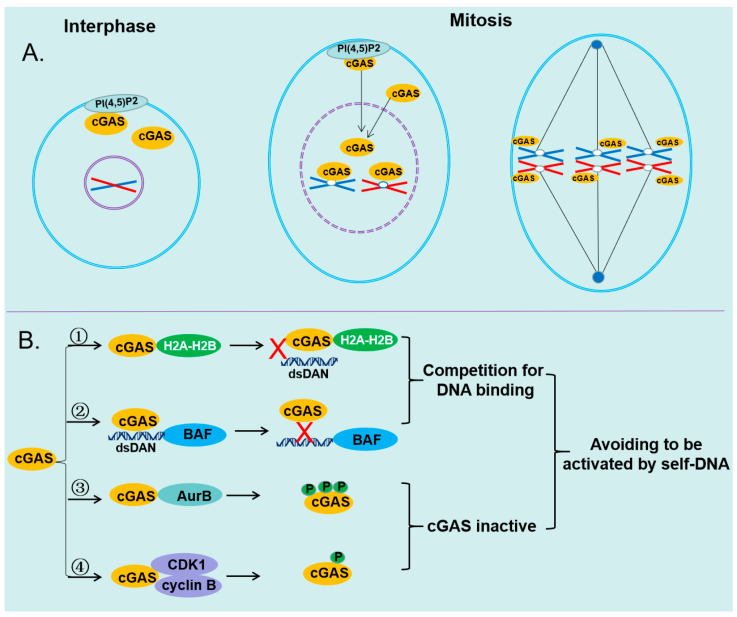
The mechanisms of cGAS avoid being activated by self-DNA. (**A**) The nuclear envelope is a highly dynamic structure that is disassembled and reassembled during mitosis in higher eukaryotes. cGAS is transported to the nucleus and interacts with chromatin during mitosis when the nuclear envelope is disassembled. (**B**) ① cGAS interacts with the H2A–H2B heterodimer. cGAS is locked in its inactive state by binding with nucleosomes, and this binding disrupts cGAS dimerization and prevents further dsDNA binding. ② BAF prevents cGAS activity by competing for DNA binding. BAF is a chromatin-binding protein essential for nuclear membrane reformation at the end of mitosis. BAF monomer has a helix-hairpin-helix DNA-binding domain, allowing BAF dimers to bridge two strands of DNA. The binding between BAF and dsDNA could cause the dissociation of cGAS from dsDNA, which dynamically displaces transiently bound cGAS monomer from dsDNA. ③ cGAS is hyperphosphorylated by AurB at the N-terminal serine and threonine residues, which inhibits the ability of cGAS to form Liquid-liquid phase condensation. ④ cGAS is phosphorylated by the CDK1-cyclin B complex at a highly conserved site Ser305, which impairs the ability of cGAS to synthesize 2′3′-cGAMP.

cGAS was first proposed as a cytosolic DNA sensor. Thus, compartmentalization of self-DNA in mitochondria and nucleus is essential for cGAS to discriminate between non-self and self-DNA. However, recent studies have indicated that cGAS is located in the cytoplasm and plasma membrane and within the nucleus [4,13]. What’s more, it has been shown that cGAS is tightly associated with chromatin during mitosis, a phase of the cell cycle when the nuclear envelope breaks down [19]. The tight association of cGAS with chromatin during mitosis raises a thought-provoking question: How cGAS activity is regulated during the cell cycle (Figure 3).

One study indicated that cGAS is tethered to the chromatin by binding to histones that form the nucleosome core, and this interaction can inhibit the activation of cGAS [52]. Another study suggested that nucleosomes could interfere with cGAS, in case cGAS should be activated by nucleosome-free regions, and nucleosomes have a higher affinity for cGAS than naked DNA to ensure it will fulfill this destiny [53]. The interaction between cGAS and the nucleosome prevents cGAS from further binding to dsDNA and abolishes the dimerization of cGAS [54]. The structure of cGAS–nucleosome complexes has shown that cGAS interacts with the H2A–H2B heterodimer through the region around site B and contacts the DNA from the adjacent nucleosome via residues within site C [54]. Thus, the dsDNA binding sites B and C are occupied by the bound nucleosomes. Although site A does not directly contact the nucleosome, it is not accessible to dsDNA due to steric hindrance. Therefore, cGAS is locked in its inactive state by the binding of nucleosomes, and this binding disrupts cGAS dimerization and prevents further dsDNA binding (Figure 3). Both the residues R236 and R255 were shown to be critical for the binding of cGAS to the nucleosomal acidic patch formed by H2A-H2B and play an important role in the inhibitory effect of nucleosomes on cGAS activity [54].

### 4.4. Barrier-to-Autointegration Factor 1 Restricts cGAS to Sense Self-DNA during Mitosis

Excepting the H2A-H2B heterodimer, the barrier-to-autointegration factor (BAF) was also shown to compete with cGAS for access to DNA in the nucleus, thereby preventing the sensing of host DNA [55]. BAF, a chromatin-binding protein essential for nuclear membrane reformation at the end of mitosis, is involved in multiple pathways, including mitosis, nuclear assembly, viral infection, chromatin and gene regulation, and the DNA damage response [56]. The unique DNA-binding properties of BAF are likely fundamental to its roles, and BAF forms homodimers, each subunit of which binds double-stranded DNA in a sequence-independent manner [56]. Each BAF monomer has a helix-hairpin-helix DNA-binding domain, through which BAF dimers bridge two strands of DNA either intra-molecularly or inter-molecularly [57].

BAF exerts an important regulatory function over cGAS, and defects in BAF can trigger innate immune activation [55]. BAF-mediated suppression of the cGAS pathway is necessary for the reactivation of Kaposi sarcoma-associated herpesvirus (KSHV) and Epstein-Barr virus (EBV) [55,58]. Inhibiting BAF expression in latently infected, reactivating, or uninfected cells leads to increased type I interferon-mediated antiviral responses and decreased viral replication [58]. Ablation of BAF by gene editing resulted in chromatin activation near host defense genes with associated increased expression of ISGs, including OAS2, Rsad2 (viperin), IFIT1, and ISG15 [58]. Down-regulation of BAF triggered a robust ISG response, whereas suppression of other relevant genes had no effect [55]. Rather than passively interfering with cGAS activity by blocking DNA binding sites, BAF dynamically displaces transiently bound cGAS monomers from dsDNA [55]. Dynamic competition by BAF at the nuclear periphery is a critical strategy the host uses to reconcile the advantages of maintaining a universal DNA recognition machinery with routine operations within a living cell [55] (Figure 3).

### 4.5. The Activity of cGAS Is Suppressed via Phosphorylation during Mitosis

cGAS is hyperphosphorylated in the nucleus, and the hyperphosphorylation can suppress the activity of cGAS, with the cGAS hyperphosphorylation mediated by Aurora kinase B (AurB), CDK1-cyclin B complex and other kinases [19,59] (Figure 3). Human cGAS is hyperphosphorylated by AurB at the N-terminal serine and threonine residues, including Ser13, Ser37, Ser64, Thr69, Thr91, Ser116, Ser129, and Ser143 [19]. Parallel reaction monitoring (PRM) showed that phosphorylation of the N-terminal serine and threonine residues in cGAS increased dramatically in mitotic cells compared with asynchronized cells [19]. Treating BJ-5ta cells with Aurora kinase inhibitors (AMG-900, MLN-8237, or AZD-1152) after cells exited the G2/M arrest, the hyperphosphorylation level of cGAS was reduced by aurora kinase inhibitors in a dose-dependent manner [19]. Like chemical inhibitors, siRNA-mediated knockdown of AurB also decreased cGAS hyperphosphorylation [19]. The hyperphosphorylation at the N-terminus could prevent the cGAS phase from separating into liquid droplets where cGAS can efficiently synthesize 2′3′-cGAMP.

Furthermore, human cGAS could be phosphorylated by the CDK1-cyclin B complex at a highly conserved site Ser305 during the mitotic phase of the cell cycle [59] (Figure 3). Phosphorylation at this site could inhibit the ability of cGAS to synthesize 2′3′-cGAMP, leading to unresponsiveness to DNA-triggered innate immunity in mitotic cells [59]. The type 1 phosphatase PP1 dephosphorylates cGAS upon mitotic exit to enable its DNA sensing ability. The human cGAS mutant (S305A) of S305 within the nuclear localization sequence (295DVIMKRKRGGS305) is localized to chromosomes in mitotic cells similarly to wild-type cGAS or cGAS (S305D); thus, phosphorylation of cGAS S305 is not required for its chromosomal localization, but preventing its activation and inhibiting the ability of cGAS to synthesize 2′3′-cGAMP during mitosis [59].

## 5. Consequences of Self-DNA Induced cGAS Activation

### 5.1. Activation of cGAS by Self-DNA Can Cause Autoimmune Diseases

Accumulated evidence has shown that the imbalance of the innate immune system was the main contributor to autoimmune diseases and acute inflammation induced by tissue damage or microbial infection [60]. Self-DNA sensed by the immune system has emerged as a key contributing response in the pathogenesis of autoimmune diseases [61]. Recently, many researchers have revealed that the regulatory role of the cGAS pathway in autoimmune diseases, including Aicardi Goutières syndrome (AGS), systemic lupus erythematosus (SLE) and Rheumatoid arthritis (RA) [58,62,63].

cGAS is required for lethal autoimmune disease in the Trex1-deficient mouse model of AGS [64]. Due to the multi-organ inflammation, Trex1-deficient mice die within a few months after birth. However, they could be rescued by deleting the gene of cGAS in TREX1-deficient [47]. Systemic lupus erythematosus (SLE) is an autoimmune disease in which pathogenic autoantibodies are produced against nucleic acids and their interacting proteins, resulting in inflammation and tissue damage [65]. Apoptosis-derived membrane vesicles (AdMVs) in SLE serum induce type I IFN (IFN-I) production through activation of the cGAS pathway [66]. The production of IFN-I could further activate immune responses that lead to tissue damage in various organs, resulting in more generation of AdMVs, triggering a positive feedback loop of IFN-I production and further tissue damage [66]. Thus, blockade of the cGAS-STING axis represents a promising therapeutic target for AGS and SLE.

### 5.2. Activation of cGAS by Self-DNA Is a Double-Edged Sword in Cancer

The ectopic cytosolic dsDNA may exert both anti-tumorigenic and pro-tumorigenic effects dependent on the specific context as well as the stage of tumor progression [67,68]. Cancer cells exhibit genomic instability and chromosomal abnormalities that commonly result in the formation of cytosolic chromatin fragments and micronuclei [69]. Subsequently, cGAS recognizes the DNA source and responds quickly to activate the downstream cascade reaction to eliminate tumor [70]. Many studies have indicated that activation of cGAS by self-DNA could improve anti-tumor immunity by enhancing tumor immune surveillance, accelerating cellular senescence, and promoting apoptosis [71,72]. The tumor cells need to evade this signaling pathway detection to survive in the harsh living environment; thus, the cGAS-STING axis was observed to be disrupted in tumors [73]. The cGAS-STING pathway suppression has been observed in colorectal carcinoma, melanoma, and cancer cells lacking telomerase [72].

However, many other studies have revealed that cGAS activation by self-DNA could promote cancer development by inducing chronic and aberrant inflammation [1,70,74]. Chronic activation of cGAS signaling in cancer cells with high chromosomal instability promotes invasion and metastasis, attributed to a switch from IFN-I and canonical NF-κB signaling to non-canonical NF-κB cascades [74]. cGAS is a major factor in promoting hepatocellular carcinoma cell (HCC) tumor growth by suppressing ferroptosis in vivo [75]. Knocking down of cGAS significantly suppressed tumor growth of Hep3B cells in a mouse xenograft model, which was ultimately rescued by the restored expression of cGAS, suggesting that the cGAS is important for tumor growth in HCC [75]. Another study indicated that tumor metastasis in mice brains was connected with the 2′3′-cGAMP transferred from tumor cells to astrocytes in an adjacent paracrine and endocytosis manner. In the process, the cGAS pathway in astrocytes was activated with IFN-α and TNF-α activation, contributing to a tumor growth advantage [76]. Thus, activating cGAS by self-DNA can carry out multiple functions in cancers.

## 6. Conclusions and Future Perspectives

cGAS is a universal DNA sensor and cannot discriminate self-DNA from non-self. Thus, both DNAs are capable of stimulating the cGAS pathway. Self-DNA sensing by the cGAS pathway can cause autoimmune diseases and regulate tumour progression. Under normal physiological conditions, cGAS avoids sensing self-DNA through the following mechanisms: DNases could degrade self-DNA under normal conditions to prevent aberrant activation of cGAS-mediated immune responses. In the meantime, cGAS is localized to the plasma membrane during the stationary phase, which could hinder it from binding self-DNA in the cytoplasm. During mitosis, cGAS is interacted with histone and BAF, which block cGAS from sensing self-DNA. Further, cGAS is hyperphosphorylated by AurB and CDK1-cyclin B complex during mitosis, suppressing the activity of cGAS.

Activation of cGAS by self-DNA could cause autoimmune diseases and regulate the progression of tumor. Therefore, targeting the cGAS-STING pathway can alleviate autoimmune symptoms and be a potential drug target for treating cancer. Many small molecule compounds, including aspirin, antimalarial drugs, RU.521, oligodeoxynucleotide containing a TTAGGG modified fragment (ODNs), RU series of compounds, suramin and PF series of compounds have been developed as the inhibitor of cGAS, which could be used as new drugs for the treatment of innate immune diseases and cancers [77,78]. However, due to the dual effects of cGAS in regulating cancer, the activation of cGAS could also exert an anti-tumor activity. Thus, several agonists of the cGAS-STING pathway were developed to treat cancers. The CDNs, including 2′3′-cGAMP, 3′5′-c-di-GMP, 3′3′-cGAMP, or CDN analogs, are ideal signaling pathway agonists. It has shown that cGAMP did not have a direct cytotoxic effect in the studied tumor cell lines in vitro, but cGAMP treatment in vivo induced apoptosis of tumor cells by possibly activating antitumor CD^8+^ T cells [79]. Additionally, several anticancer therapies designed to target cancer cells directly are now shown to indirectly activate the cGAS-STING pathway, which induces anti-tumor immunity [80,81]. It has verified that radiation, chemotherapy, heat-inactivated modified vaccinia virus and listeria monocytogenes therapeutic vaccines could indirectly activate the cGAS-STING pathway, which could induce the antitumor immune responses [81,82].

cGAS-STING-TBK1-IRF3 signal axis is the main pathway of cGAS-inducing interferon and inflammatory responses. It has shown that not only the activity of cGAS was suppressed during mitosis. Indeed, the phosphorylations of STING and IRF3 were also inhibited during mitosis by an unknown mechanism [83]. A study from human keratinocytes has indicated that the phosphorylations of STING and IRF3 could not be stimulated by transfection of exogenous cGAMP in cells arrested at the prometaphase [83]. In mammalian cells, the Golgi complex comprises stacks connected by membranous tubules. During mitosis, the Golgi complex is disassembled into isolated stacks, which causes dispersion of the Golgi apparatus throughout the cytoplasm [84]. Since the activation of the cGAS-STING-TBK1-IRF3 signal axis requires the transporting of STING from ER to the Golgi, the Golgi dispersion may restrict the cGAS-STING pathway. Thus, the effects of Golgi dispersion in the cGAS-STING pathway during mitosis should be investigated in future.

Further, cGAS was shown as a shuttle protein transported among the plasma membrane, cytoplasm and nucleus [69,85]. The transportation of cGAS is dependent on several factors, including pathogenic infection, cell cycle and DNA damage. The multiple functions of cGAS depend on its subcellular localization. On the plasma membrane, cGAS could be prevented to recognize self-DNA. In the cytoplasm, cGAS could sense DNA and trigger IFN and inflammation responses. Transporting to the nucleus, the activity of cGAS could be inhibited through interacting with histones. However, the exact regulatory mechanism of cGAS transportation is still unclear. Thus, it is recommended that studies should be designed to explore the mechanism of cGAS transportation.

Moreover, the cGAS signaling plays an integral role in the host immune response, and the activation of cGAS is highly related to various autoimmune diseases and cancers. Therefore, targeting the cGAS pathway has become a promising strategy in the therapy of autoimmune diseases and cancers. Indeed, many agonists or inhibitors of the cGAS pathway have been tested in clinical trials for autoimmune diseases and cancer immunotherapy. Besides the direct inhibition of the cGAS signaling, targeting the downstream nodes of the cGAS signaling could also exert opposite effects on autoimmune diseases. It has been suggested that inhibiting the TBK1 signaling by using BX795 could down-regulate IFN-I activation in PBMCs of Sjögren’s Syndrome (SS), SLE, and multiple sclerosis (MS) patients [86]. Therefore, targeting downstream nodes of the cGAS signaling is also an alternative strategy for treating autoimmune diseases and cancers. Future studies are necessary to explore the effects of modulators targeting the downstream nodes of the cGAS signaling in autoimmune diseases and cancers. Additionally, due to its indirect regulation and dual roles affecting diverse downstream regulatory factors, cGAS exerts a dichotomous effect on tumors after sensing self-DNA. Therefore, the activation status of cGAS and its function in a particular tumor should be considered when an intervention method targeting the cGAS pathway for cancer therapy is developed.

## 7. Methodology

This is a narrative review based on an existing literature search. This review focuses on how cGAS avoid sensing self-DNA under normal physiological conditions. We included original research or review articles published in the last ten years. We only had studies written in English. We started the literature search by using the keywords “cGAS senses self-DNA”, “cGAS avoids sensing self-DNA”, “cGAS is activated by self-DNA”, “self-DAN induces autoimmune diseases through cGAS”, “self-DNA induces cancers through cGAS”, “the cGAS-STING pathway in the treatment of autoimmune diseases”, “the cGAS-STING pathway in the treatment of cancers”.

We searched articles by using PubMed (https://www.ncbi.nlm.nih.gov (accessed on 1 September 2022 to 27 September 2023)). Due to the development of science and technology, we limit the publication date to 2013 and 2023 (articles published in the past ten years) so that we can summarize the information based on the recent literature. For each manuscript, preliminary relevance was determined by title. From the title, if the content seemed to discuss the relation between cGAS and self-DNA, we obtained its full information for further evaluation. We skimmed through the full-text articles to further evaluate the quality of the studies. A total of 225 studies were deemed relevant, and we obtained the full-text article for quality assessment.

From each study, we extracted information on the following subtopics: (1) Structural domains and modification sites of cGAS. (2) The mechanisms of cGAS activated by DNA. (3) The mechanisms of cGAS avoid sensing self-DNA. (4) Consequences of self-DNA induced cGAS activation. (5) The role of the cGAS-STING pathway in the treatment of autoimmune diseases and cancers.

## Data Availability

The data presented in this study are available in the insert article.
